# Predictive performance of two types of urinary biomarkers for renal non-recovery in sepsis-associated acute kidney injury: a prospective observational study

**DOI:** 10.1186/s12882-024-03589-9

**Published:** 2024-05-03

**Authors:** Licheng, Huimiao Jia, Yijia Jiang, Wenxiong Li

**Affiliations:** 1 Department of Emergent Intensive Critical Unit, Beijing Lu-He Hospital, Capital Medical University, Beijing, 101100 China; 2 Department of Surgical Intensive Critical Unit, Beijing Chao-yang Hospital, Capital Medical University, 8 GongrenTiyuchangNanlu, Chaoyang District, Beijing, 100020 China

**Keywords:** C-C motif chemokine ligand 14, [TIMP-2]•[IGFBP7], sepsis, Acute kidney injury

## Abstract

**Background and purpose:**

Renal non-recovery is known to have negative prognostic implications in patients suffering from acute kidney injury (AKI). Nevertheless, the identification of biomarkers for predicting renal non-recovery in sepsis-associated AKI (SA-AKI) within clinical settings remains unresolved. This study aims to evaluate and compare the predictive ability for renal non-recovery, use of kidney replacement therapy (KRT) in the Intensive Care Unit (ICU), and 30-day mortality after SA-AKI by two urinary biomarkers, namely C-C motif chemokine ligand 14 (CCL14) and [TIMP-2]•[IGFBP7].

**Methods:**

We prospectively screened adult patients who met the criteria for AKI stage 2–3 and Sepsis-3.0 in two ICUs from January 2019 to May 2022. Patients who developed new-onset SA-AKI after ICU admission were enrolled and urinary biomarkers including [TIMP-2]•[IGFBP7] and CCL14 were detected at the time of SA-AKI diagnosis. The primary endpoint was non-recovery from SA-AKI within 7 days. The secondary endpoints were the use of KRT in the ICU and 30-day mortality after SA-AKI. The individual discriminative ability of [TIMP-2]•[IGFBP7] and CCL14 to predict renal non-recovery were evaluated by the area under receiver operating characteristics curve (AUC).

**Results:**

141 patients with stage 2–3 SA-AKI were finally included, among whom 54 (38.3%) experienced renal non-recovery. Urinary CCL14 exhibited a higher predictive capability for renal non-recovery compared to [TIMP-2]•[IGFBP7], with CCL14 showing an AUC of 0.901, versus an AUC of 0.730 for [TIMP-2]•[IGFBP7] (*P* = 0.001). Urinary CCL14 and [TIMP-2]•[IGFBP7] demonstrated a moderate predictive value for the need for KRT in ICU, with AUC values of 0.794 and 0.725, respectively; The AUC of [TIMP-2]•[IGFBP7] combined with CCL14 reached up to 0.816. Urinary CCL14 and [TIMP-2]•[IGFBP7] exhibited poor predictive power for 30-day mortality, with respective AUC values of 0.623 and 0.593.

**Conclusion:**

Urinary CCL14 had excellent predictive value for renal non-recovery in SA-AKI patients. For predicting the use of KRT in the ICU, the predictive capability of urinary [TIMP-2]•[IGFBP7] or CCL14 was fair. However, a combination of [TIMP-2]•[IGFBP7] and CCL14 showed good predictive ability for the use of KRT.

**Supplementary Information:**

The online version contains supplementary material available at 10.1186/s12882-024-03589-9.

## Background

Sepsis, a frequently encountered condition within the intensive care unit (ICU), frequently precipitates organ dysfunction and poses life-threatening complications [1]. Among these complications, acute kidney injury (AKI) emerges as a prevalent concern, exhibiting an incidence rate ranging from 40 to 50% [2]. AKI is characterized by deterioration in renal function, leading to impaired regulation of extracellular volume and clearance of circulating substances [3]. This ultimately contributes to the development of chronic kidney disease (CKD) and cardiovascular events. The impact of AKI on morbidity and mortality is substantial, often resulting in an unfavorable prognosis. It is widely acknowledged that the patient’s prognosis is influenced by the severity and duration of AKI [4]. Notably, even a failure to recover renal function within 48 h can significantly affect the patient’s prognosis, exerting both short-term and long-term consequences [5, 6].

Sepsis induces renal hemodynamic abnormalities, triggers immune cell activation, releases inflammatory mediators, and suppresses endogenous hormone production, thereby impacting both the glomerulus and renal tubules [7]. The presence of sepsis-associated AKI (SA-AKI) is associated with elevated mortality rates and an augmented incidence of long-term complications [1, 8]. Damaged renal tubules can release biomarkers: tissue inhibitor of metalloproteinase-2 (TIMP-2) is primarily secreted and expressed by distal tubular cells [9], while insulin-like growth factor-binding protein 7 (IGFBP7) is expressed throughout the renal tubules but is mainly secreted by proximal tubular cells [10]. Previous studies have reported that [TIMP-2]•[IGFBP7] (the product of TIMP-2 and IGFBP7) has shown significant predictive ability for the progression of AKI (stages 2–3) within 12 h of sample collection in patients with various diseases, including sepsis [11]. CCL14, a member of the chemokine family, exhibits consistent expression in diverse tissues, including the kidney. Its involvement in pro-inflammatory chemotaxis in multiple diseases is noteworthy, as it activates monocytes and macrophages [12]. Furthermore, CCL14 serves as a novel third-generation biomarker for assessing the risk of renal non-recovery in cases of AKI lasting for 3 days or more [13]. In a comprehensive international cohort study involving medical and surgical intensive care patients, several potential biomarkers were discovered to have predictive value for persistent KDIGO stage 3 AKI lasting for 72 h or more. Among these biomarkers, urinary CCL14 exhibited the highest effectiveness [14]. Our prior investigation revealed that both urinary [TIMP-2]•[IGFBP7] and CCL14 have useful predictive value for renal non-recovery from AKI in such patients; however, CCL14 did not demonstrate a significant advantage over [TIMP-2]•[IGFBP7] in forecasting renal non-recovery [15].

To compare the predictive value of urinary [TIMP-2]•[IGFBP7] and CCL14 for renal non-recovery and to ensure the homogeneity of the included population, this prospective observational study aimed to evaluate and compare the predictive abilities of the urinary [TIMP-2]•[IGFBP7] and CCL14 for renal non-recovery within 7 days, as well as the necessity for KRT in ICU and 30-day mortality. The focus was specifically on ICU patients with Stage 2–3 SA-AKI.

## Materials and methods

### Study Design and patients

**Fig. 1:**
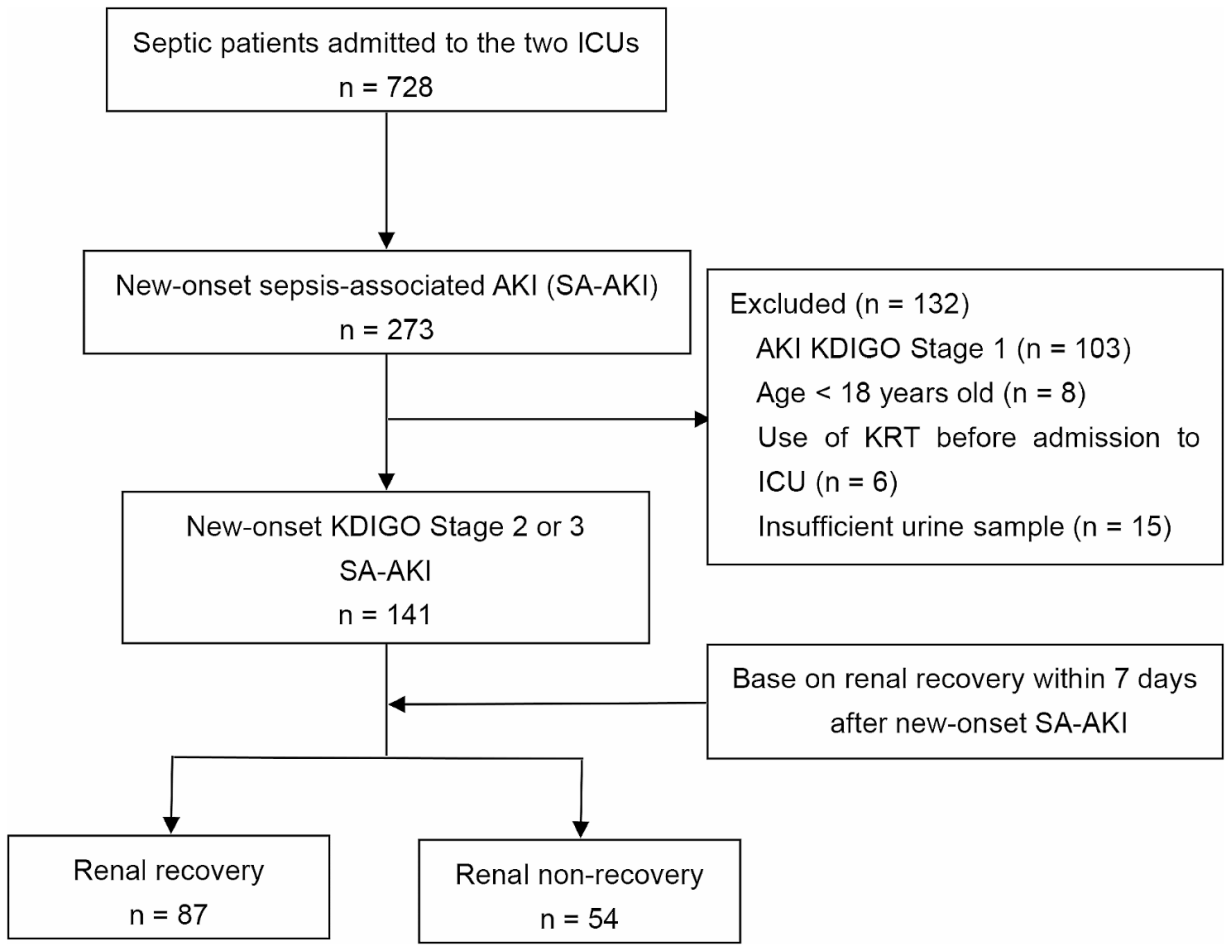
Study flow diagram. *Abbreviations*: ICU, intensive care unit; AKI, acute kidney injury; KDIGO, Kidney Disease: Improving Global Outcomes; RRT, Renal Replacement Therapy; SA-AKI, sepsis-associated acute kidney injury

This prospective, observational study was conducted from January 2019 to May 2022 at two tertiary hospitals in China. Approval for the study protocol was obtained from the Ethics Committee of Beijing Chao-Yang Hospital (ethics number 2018 − 117). The study followed the ethical principles of the Declaration of Helsinki 1964. Informed consent from patients or their next of kin was obtained before patients joined in the study. We prospectively screened patients from two ICUs (Fig. 1). Adult patients were eligible for inclusion if they stayed longer than 24 h in ICU, and met the criteria for AKI stage 2–3 and the Sepsis-3.0. None of the following exclusion criteria was fulfilled: (1) age < 18 years old; (2) patients with KRT use before admission to the ICU; (3) existing AKI before admission to the ICU; (4) insufficient urine samples.

### Data collection and biomarkers measurement

At the time of admission to the intensive care unit (ICU), demographic, clinical, and laboratory data, as well as prior health history and chronic comorbidities, were gathered. On the day of the AKI onset, Sequential Organ Failure Assessment (SOFA) score and Acute Physiology and Chronic Health Evaluation (APACHE II) score were evaluated. Serum creatinine (SCr) levels were measured and documented upon ICU admission and every 12 h until 7 days following the onset of AKI. Additionally, the hourly urine volume was measured and recorded during the ICU period. Furthermore, information regarding utilization of KRT in ICU, mortality during the ICU stay, duration of hospitalization, and 30-day mortality after the diagnosis of AKI were collected.

Urine samples were obtained upon the diagnosis of AKI. These samples underwent centrifugation at a speed of 3000 revolutions per minute for 10 min. The resulting supernatant urine was extracted and stored at a temperature of -80 °C for subsequent analysis. The concentration of [TIMP-2] and [IGFBP7] were measured using a commercially available NephroCheck Test (Astute Medical, San Diego, CA, USA). Additionally, CCL14 levels were quantified through the utilization of an enzyme-linked immunosorbent assay (ab272201, Abcam, UK) by investigators who were unaware of the patient’s information. The VITROS 5600 Integrated System reports the product of the two protein concentrations ([TIMP-2]•[IGFBP7]) in units of (ng/mL)^2^/1000.

### Clinical outcomes

The primary outcome of the study was the occurrence of renal non-recovery within a 7-day period following SA-AKI. The secondary outcomes examined included use of KRT in the ICU and 30-day mortality after SA-AKI. Patients who met at least one of the following five indications were given KRT: (1) The presence of acute severe pulmonary edema that did not respond to diuretic therapy. (2) PH below 7.15 in the context of pure metabolic acidosis which was refractory to medical treatment. (3) Serum potassium concentration exceeding 6.5 mmol/L which was refractory to medical treatment. (4) Serum urea nitrogen exceeding 112 mg/dL (40 mmol/L). (5) Oliguria or anuria for more than 72 h [16, 17].

### Definitions

Sepsis, a condition characterized by an uncontrolled host response to infection resulting in life-threatening organ dysfunction, has been defined by the Third International Consensus as Sepsis and Septic Shock (Sepsis-3) [18].

AKI is diagnosed based on the criteria established by KDIGO guidelines, meeting any of the following: (1) increase in serum creatinine (SCr) ≥ 0.3 mg/dL (≥ 26.5 µmol/L) within 48 h; (2) increase in SCr to ≥ 1.5 times baseline, which was known or suspected to have occurred within 7 days in the past; (3) urine output (UO) < 0.5 ml/kg/h for more than 6 h. AKI classification criteria were defined as follows: (1) stage 1: 1.5–1.9 times increase of SCr relative to baseline, or increase in SCr ≥ 0.3 mg/dL within 48 h, or UO < 0.5 ml/kg/h for 6–12 h; (2) stage 2: 2.0–3.0 times increase of SCr relative to baseline, or UO < 0.5 ml/kg/h for ≥ 12 h; (3) stage 3: > 3.0 times increase of SCr relative to baseline, or increase in SCr to ≥ 4.0 mg/dL (≥ 353.6 mmol/L), or initiation of KRT (regardless of the change in SCr) or UO < 0.3 ml/kg/h for ≥ 24 h, or anuria for ≥ 12 h [19].

The baseline creatinine was defined as follows: if at least five values were available the median of all values available from 6 months to 6 days before enrollment was used. Otherwise, the lowest value in the 5 days before enrollment was used. If no pre-enrollment creatinine was available or the emergency patient’s serum creatinine was abnormal at the time of admission, the baseline creatinine was estimated using the Modification of Diet in Renal Disease (MDRD) equation assuming that baseline eGFR is 75 ml/min per 1.73 m^2^ [11].

The criteria to identify patients with stage 2–3 SA-AKI is characterized by the simultaneous presence of sepsis, as defined in adults according to the Sepsis-3 criteria, and stage 2–3 AKI, as defined by the KDIGO criteria [20]. Renal recovery is operationally defined as the absence of any stage of AKI based on the criteria of either SCr or urine output. Specifically, individuals diagnosed with AKI stage 2 must demonstrate a reduction in SCr to less than 150% of their baseline level and maintain a urine output exceeding 0.5 ml/kg/h for a duration exceeding 6 h. Patients who necessitate KRT or succumb to mortality within 7 days following AKI are classified as experiencing renal non-recovery [21].

### Statistical analysis

Continuous variables were expressed as mean ± standard deviation (SD) or median (interquartile range, IQR), depending on their data distribution. Group comparisons were performed using either a t-test or the rank-sum test. Categorical variables were presented as numbers (n) and percentages (%), and group comparisons were conducted using the chi-square (χ^2^) test or Fisher’s exact test.

The determinants of renal non-recovery in patients with SA-AKI were investigated using multivariate logistic regression analysis, which was performed with a stepwise forward-selection process. Univariate analysis initially assessed a spectrum of clinical parameters commonly associated with renal non-recovery, including age, serum lactate levels, sepsis severity, SCr at enrollment, and AKI severity. Only those clinical parameters that achieved statistical significance (*P* < 0.05) in the univariate analysis were carried forward into the multivariate logistic regression analysis. Ordinal variables were included in the regression analysis without modification. Continuous variables were converted into categorical entities and were employed in the regression model as dummy variables to facilitate the analysis. Variables with *P* < 0.05 in the multivariate logistic regression were independent risk factors for renal non-recovery.

We assessed the predictive capacity of various biomarkers in determining renal non-recovery by employing the area under the receiver-operating characteristic (ROC) curve (AUC). The following values were used to describe the AUC: 0.90–1.0, excellent; 0.80–0.89, good; 0.70–0.79, fair; 0.60–0.69, poor; and 0.50–0.59, useless. To evaluate the efficacy of various cutoff values of urinary biomarkers in predicting renal non-recovery, ROC curve analysis was conducted. The optimal cutoff point is identified by finding a balance point that maximizes the sum of sensitivity and specificity. This balance point ensures that the test is as accurate as possible in both identifying patients who will not recover renal function and those who will recover. By maximizing the sum of sensitivity and specificity, the optimal cutoff point serves as a threshold for the most accurate prediction of renal non-recovery. Subsequently, the optimal cut-off values of [TIMP-2]•[IGFBP7] and CCL14 were utilized to estimate Kaplan-Meier survival curves and compare them using the Log-rank test for 30-day mortality following the onset of SA-AKI. Statistical analyses were performed using SPSS Statistics 24, R 3.6.1, and MedCalc software. A significance level of *P* < 0.05 was deemed statistically significant.

## Results

### Baseline characteristics of patients

**Fig. 2:**
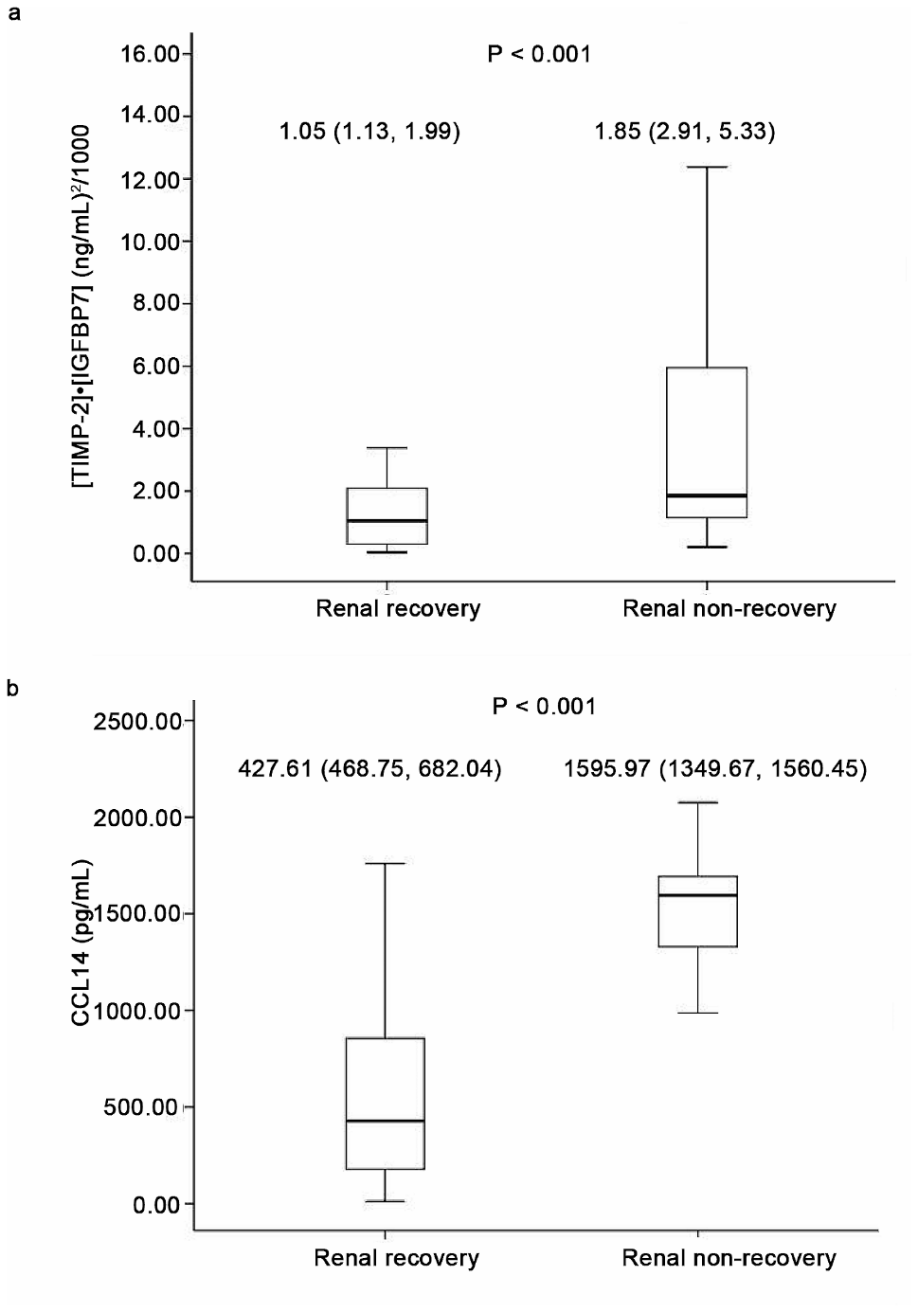
Urinary [TIMP-2]•[IGFBP7] and CCL14 levels at AKI diagnosis in renal recovery and renal non-recovery group. a: The comparison of urinary [TIMP-2]•[IGFBP7] levels of renal recovery and non-recovery. b: The comparison of urinary CCL14 levels of renal recovery and non-recovery. *Abbreviations*: SA-AKI, sepsis-associated acute kidney injury; TIMP-2, Tissue inhibitor of metalloproteinase-2; IGFBP7, Insulin-like growth factor-binding protein 7; CCL14, C-C motif chemokine ligand 14

During the designated study period, a total of 728 sepsis patients were initially screened. Among them, 273 individuals were identified as having newly developed SA-AKI. Subsequently, 132 patients were excluded from the study for various reasons, including 103 patients who exhibited AKI at KDIGO Stage 1, 8 patients who were under the age of 18, 6 patients who had previously undergone KRT before their admission to the ICU, and 15 patients who had insufficient urine samples. Ultimately, a cohort of 141 patients was enrolled for analysis (Fig. 1), and their baseline characteristics are displayed in Table 1. Out of 141 patients, 54 did not show renal recovery. Among these, 31 patients were classified as having renal non-recovery based on the established criterion for renal non-recovery. Additionally, 11 patients were categorized as renal non-recovery due to dependence on KRT at day 7, and 12 patients were defined as non-recovery due to death at day 7. These patients with renal non-recovery exhibited a significantly higher APACHE II score (18 vs. 18.5, *P* = 0.018), a higher non-renal SOFA score (8.0 vs. 6.0, *P* = 0.020), a higher prevalence of septic shock (31.5% vs. 13.8%, *P* = 0.012), higher initial SCr levels at the onset of AKI (2.55 mg/dL vs. 1.69 mg/dL, *P* < 0.001), and elevated serum lactate levels (3.7 mmol/L vs. 2.3 mmol/L, *P* = 0.002) in comparison to those who experienced renal recovery. Figure 2 showed urinary concentrations of [TIMP-2]•[IGFBP7] and CCL14 at the time of AKI diagnosis; the concentrations of [TIMP-2]•[IGFBP7] and CCL14 were observed to be higher in the non-recovery group compared to the recovery group. The [TIMP-2]•[IGFBP7] concentration was 1.85 (ng/mL)^2^/1000 versus 1.05 (ng/mL)^2^/1000 (*P* < 0.001), and the CCL14 concentration was 1595.97 pg/mL versus 427.61 pg/mL (*P* < 0.001), respectively.

**Table 1:** Baseline characteristics between patients with renal recovery and non-recovery

Variables	Renal recovery *n* = 87	Renal non-recovery *n* = 54	*p*-value
Patient characteristics			
Age (years)	60 ± 17	59 ± 18	0.658
Male	55 (63.2)	32 (59.3)	0.638
Body mass index (kg/m^2)^	24.4 ± 4.3	25.6 ± 3.7	0.079
APACHE II score	18.0 (15.8, 19.3)	18.5 (18.7, 23.2)	0.018
Non-renal SOFA score	6.0 (5.5, 7.4)	8.0 (6.8, 9.3)	0.020
Baseline SCr (mg/dL)	0.72 (0.70, 0.78)	0.76 (0.74, 0.81)	0.057
SCr at enrollment (mg/dL)	1.69 (1.50, 2.53)	2.55 (2.94, 4.70)	< 0.001
Chronic comorbidities			
Chronic kidney disease	7 (8.0)	7 (13.0)	0.343
Hypertension	31 (47.1)	30 (55.6)	0.330
Diabetes	26 (29.9)	19 (35.2)	0.512
Coronary artery disease	21 (24.1)	6 (11.1)	0.056
COPD	5 (5.7)	5 (9.3)	0.507
Chronic liver disease	24 (27.6)	10 (18.5)	0.221
Sepsis severity			0.012
Sepsis	75 (86.2)	37 (68.5)	
Septic shock	12 (13.8)	17 (31.5)	
Admission type			
Surgical	18 (21.4)	8 (14.8)	0.332
Medical	53 (60.9)	32 (59.3)	0.845
Emergency	16 (18.4)	14 (25.9)	0.288
Characteristics at inclusion			
Hemoglobin (g/L)	102.0 (100.1, 112.6)	106.5 (89.4, 108.1)	0.240
Lactate (mmol/L)	2.3 (2.9, 4.4)	3.7 (4.6, 8.2)	0.002
AKI stage at enrollment			< 0.001
Stage 2	69 (79.3)	15 (34.1)	
Stage 3	18 (20.7)	29 (65.9)	
Mechanical ventilation	74 (85.1)	43 (79.6)	0.404
Use of diuretics	38 (43.7)	29 (53.7)	0.247

*Abbreviations* AKI, acute kidney injury; APACHE, Acute Physiology and Chronic Health Evaluation; COPD, chronic obstructive pulmonary disease; SCr, serum creatinine; SOFA, Sequential Organ Failure Assessment.

### Clinical outcomes

Table 2 presents the clinical outcomes, revealing a significantly higher rate of KRT use (40.7% vs. 19.5%, *P* = 0.006) in the renal non-recovery group compared to the recovery group. Additionally, the renal non-recovery group experienced a longer hospital stay (20.5 days vs. 17.0 days, *P* = 0.028), higher ICU mortality (22.2% vs. 9.2%, *P* = 0.031), higher hospital mortality (29.6% vs. 14.9%, *P* = 0.036), and higher 30-day mortality (39.2% vs. 19.3%, *P* = 0.011). However, there was no significant difference in the length of ICU stay between the renal recovery and non-recovery groups (10 days vs. 7.0 days, *P* = 0.170).

**Table 2:** Clinical outcomes between patients with renal recovery and non-recovery

Variables	Renal recovery *n* = 87	Renal non-recovery *n* = 54	*p*-value
KRT use in ICU	17 (19.5)	22 (40.7)	0.006
ICU stay (days)	7.0 (8.0, 11)	10.0 (10, 16)	0.170
Hospital stay (days)	17.0 (16.2, 19.8)	20.5 (22.6, 43.2)	0.028
ICU mortality	8 (9.2)	12 (22.2)	0.031
Hospital mortality	13 (14.9)	16 (29.6)	0.036
30-day mortality	16 (19.3)	20 (39.2)	0.011

*Abbreviations* KRT, kidney replacement therapy; ICU, Intensive Care Unit.

### Risk factors associated with renal non-recovery

Univariate logistic regression analysis demonstrated significant associations between renal non-recovery and several factors, including age, sepsis severity, serum lactate level, SCr at enrollment, AKI stage, as well as urinary levels of [TIMP-2]•[IGFBP7] and CCL14 (all with *P* < 0.05). Subsequent multivariate logistic regression analysis (variables with *P* < 0.05 in the univariate logistic regression analysis) was conducted to evaluate the risk factors influencing renal non-recovery. This analysis confirmed that elevated urinary [TIMP-2]•[IGFBP7] (with an odds ratio [OR] of 1.261, 95% confidence interval [CI] of 1.063–1.495, and *P* = 0.008) and CCL14 (OR 1.003, 95% CI 1.002–1.004, *P* < 0.001) are independent predictors of increased risk for renal non-recovery (Table 3).

**Table 3:** Risk factors analysis for renal non-recovery

Variables	Univariate			Multivariate		
	OR	95% CI	*p*- value	OR	95% CI	*p*-value
Sepsis severity						
Sepsis	1 [Reference]					
Septic shock	2.765	1.210–6.319	0.016			
SCr at enrollment	1.005	1.002–1.008	0.003			
Serum lactate	1.119	1.036–1.209	0.004			
AKI stage at enrollment						
Stage 2	1 [Reference]					
Stage 3	4.447	2.111–9.368	< 0.001			
[TIMP-2]•[IGFBP7]	1.337	1.151–1.554	< 0.001	1.261	1.063–1.495	0.008
CCL14	1.003	1.002–1.004	< 0.001	1.003	1.002–1.004	< 0.001

*Abbreviations* AKI, acute kidney injury; CCL14, C-C motif chemokine ligand 14; CI, confidence interval; IGFBP-7, insulin-like growth factor-binding protein 7; OR, odds ration; SCr, serum creatinine; TIMP-2, tissue inhibitor of metalloproteinases-2.

### [TIMP-2]•[IGFBP7] and CCL14 as biomarkers for the prediction of renal non-recovery

**Fig. 3:**
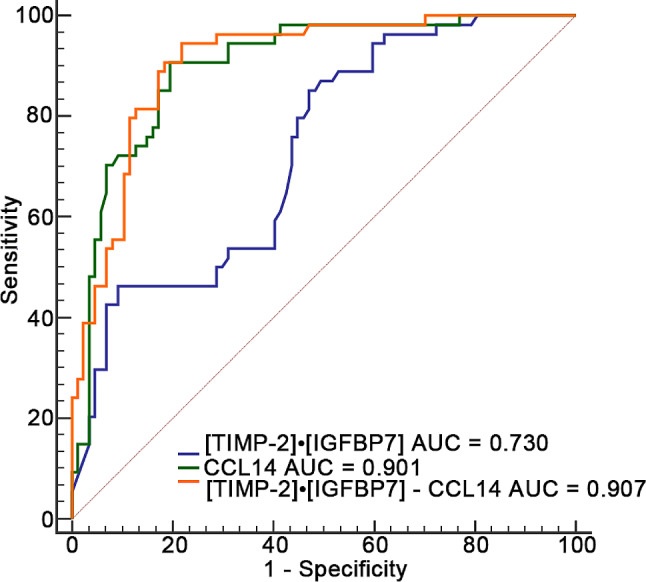
The predictive value of [TIMP-2]•[IGFBP7], CCL14 at AKI diagnosis and combined model for renal non-recovery. AUCs of [TIMP-2]•[IGFBP7], CCL14, and combined model( [TIMP-2]•[IGFBP7] - CCL14) for renal non-recovery. *Abbreviations*: ROC, receiver operating characteristic; AUC, the area under the curve; AKI, acute kidney injury; SA-AKI, sepsis-associated acute kidney injury; TIMP-2, Tissue inhibitor of metalloproteinase-2; IGFBP7, Insulin-like growth factor-binding protein 7; CCL14, C-C motif chemokine ligand 14

ROC curves were utilized to compare the predictive efficacy of [TIMP-2]•[IGFBP7] and CCL14 concerning renal non-recovery (as shown in Table 4; Fig. 3). The AUC analysis revealed that urinary levels of CCL14 exhibited a superior predictive capacity for renal non-recovery when compared to [TIMP-2]•[IGFBP7] (0.901 vs. 0.730, *P* = 0.001) (as indicated in Table S1). The threshold for CCL14 in predicting renal non-recovery was determined to be 973.95 pg/mL, yielding sensitivity and specificity rates of 90.7% and 80.5% respectively. Similarly, the threshold for [TIMP-2]•[IGFBP7] was established at 1.07 (ng/mL)^2^/1000, resulting in sensitivity and specificity rates of 85.2% and 52.9% respectively. Furthermore, When [TIMP-2]•[IGFBP7] was combined with CCL14, there was an increase in the AUC to 0.907, indicating enhanced predictive accuracy for renal non-recovery. This combined biomarker approach yielded a high sensitivity of 94.4% and a satisfactory specificity of 78.2%. However, the predictive contributions of CCL14 and [TIMP-2]•[IGFBP7] - CCL14 were not found to be statistically significant as evidenced by Delong analysis (*P* = 0.641), as shown in Table S1.

**Table 4:** The areas under the receiver operating characteristic curves for two kinds of urinary biomarkers to predict renal non-recovery

Variables	AUC	95% CI	*P*- value	Cut-off value	Sensitivity	Specificity
[TIMP-2]•[IGFBP7] ((ng/mL)^2^/1000)	0.730	0.649–0.801	< 0.001	1.07	85.2	52.9
CCL14 (pg/mL)	0.901	0.839–0.945	< 0.001	973.95	90.7	80.5
[TIMP-2]•[IGFBP7] - CCL14	0.907	0.847–0.950	< 0.001	0.252	94.4	78.2

*Abbreviations* AUC, area under the receiver operating characteristic; CCL14, C-C motif chemokine ligand 14; CI, confidence interval; IGFBP-7, insulin-like growth factor-binding protein 7; TIMP-2, tissue inhibitor of metalloproteinases-2.

### [TIMP-2]•[IGFBP7] and CCL14 as biomarkers for the prediction of KRT in ICU after AKI

**Fig. 4:**
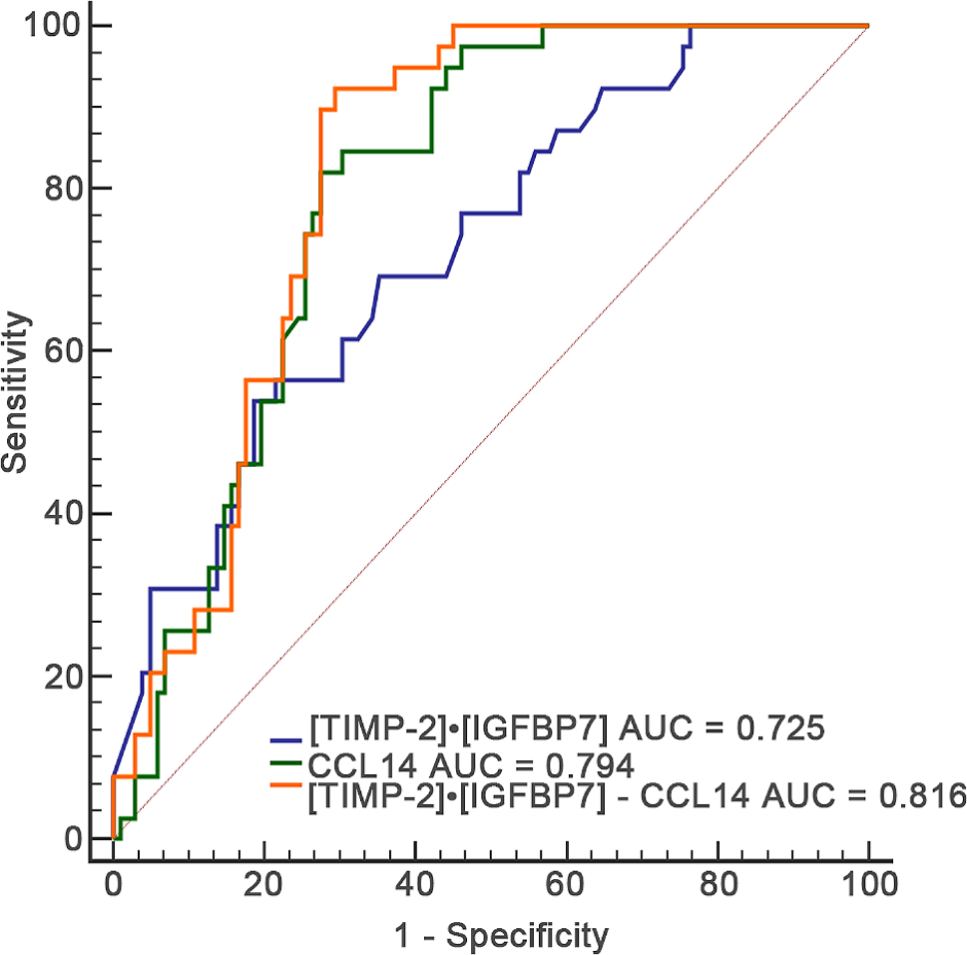
The predictive value of [TIMP-2]•[IGFBP7], CCL14 and combined model for KRT use in ICU. AUCs of [TIMP-2]•[IGFBP7], CCL14 and combined model ([TIMP-2]•[IGFBP7] - CCL14) for KRT use. *Abbreviations*: ROC, receiver operating characteristic; AUC, the area under the curve; AKI, acute kidney injury; KRT, Kidney Replacement Therapy; TIMP-2, Tissue inhibitor of metalloproteinase-2; IGFBP7, Insulin-like growth factor-binding protein 7; CCL14, C-C motif chemokine ligand 14

ROC curves were utilized to compare the predictive value of [TIMP-2]•[IGFBP7] and CCL14 for the occurrence of KRT in the ICU following AKI, as presented in Table 5; Fig. 4. The AUC values indicated that the urinary levels of CCL14 and [TIMP-2]•[IGFBP7] exhibited moderate predictive power for the prediction of KRT in the ICU after SA-AKI, with AUC values of 0.794 (95% CI, 0.718–0.858) and 0.725 (95% CI, 0.643–0.796) (*P* = 0.230), respectively, as shown in Table S2. In addition, the AUC of [TIMP-2]•[IGFBP7] combined with CCL14 reached up to 0.816, with a sensitivity of 92.3% and a specificity of 70.6%. The determined threshold for CCL14 in predicting KRT in the ICU after SA-AKI was 1073.61 pg/mL; the sensitivity and specificity values of CCL14 were 82.1% and 72.5%, respectively. the threshold for [TIMP-2]•[IGFBP7] was set at 2.46 (ng/mL)^2^/1000, with its sensitivity and specificity being 53.8% and 81.4%, respectively.

**Table 5:** The areas under the receiver operating characteristic curves for urinary biomarkers to predict use of kidney replacement therapy

Variables	AUC	95% CI	*P*-value	Cut-off value	Sensitivity	Specificity
[TIMP-2]•[IGFBP7] ((ng/mL)^2^/1000)	0.725	0.643–0.796	< 0.001	2.46	53.8	81.4
CCL14 (pg/mL)	0.794	0.718–0.858	< 0.001	1073.61	82.1	72.5
[TIMP-2]•[IGFBP7] - CCL14	0.816	0.742–0.876	< 0.001	0.232	92.3	70.6

*Abbreviations* AUC, area under the receiver operating characteristic; CCL14, C-C motif chemokine ligand 14; CI, confidence interval; IGFBP-7, insulin-like growth factor-binding protein 7; TIMP-2, tissue inhibitor of metalloproteinases-2.

### The correlation between [TIMP-2]•[IGFBP7] and CCL14 with 30-day mortality after AKI

**Fig. 5:**
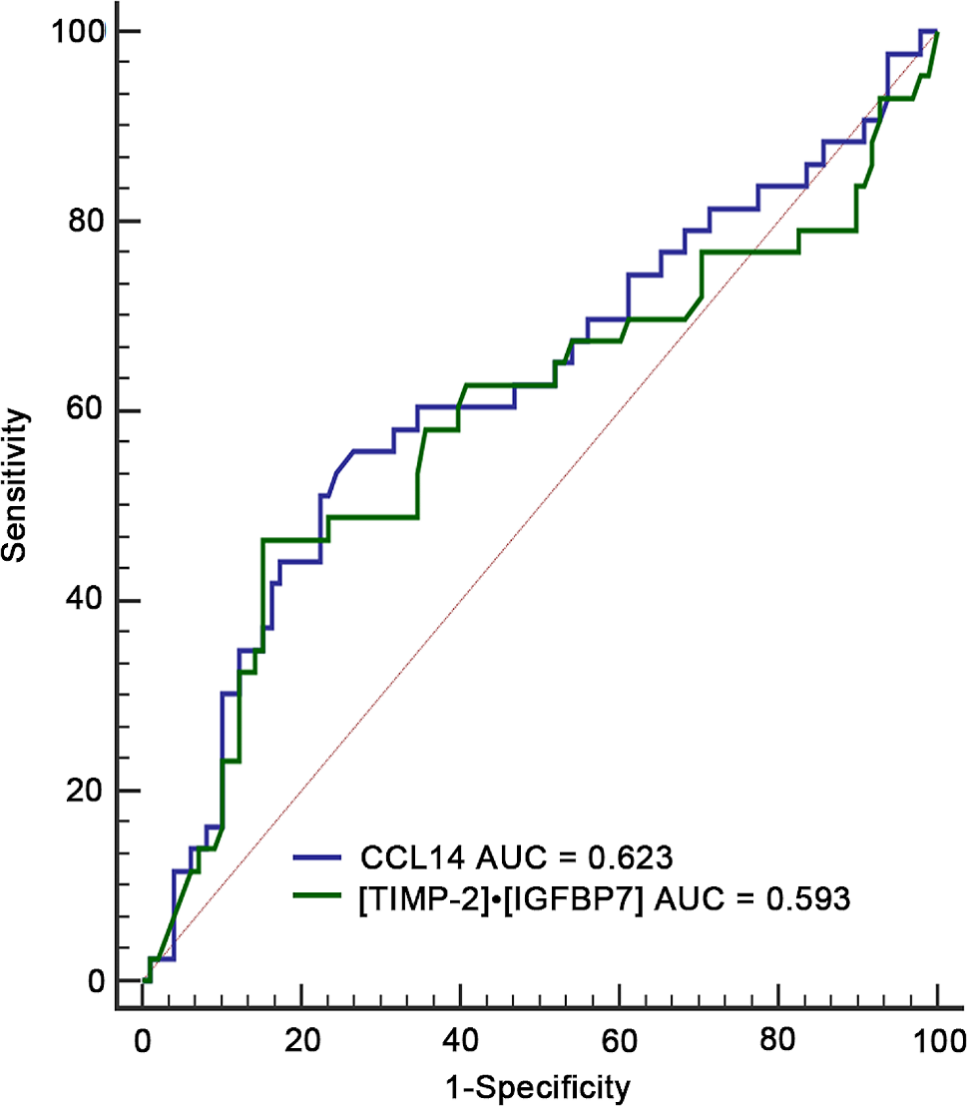
The predictive value of biomarkers for 30-day mortality after AKI. The ROC curves of urinary [TIMP-2]•[IGFBP7] and CCL14 for predicting 30-day mortality after AKI. *Abbreviations*: ROC, receiver operating characteristic; AUC, area under the ROC. TIMP-2, Tissue inhibitor of metalloproteinase-2; IGFBP7, Insulin-like growth factor-binding protein 7; CCL14, C-C motif chemokine ligand 14

**Fig. 6:**
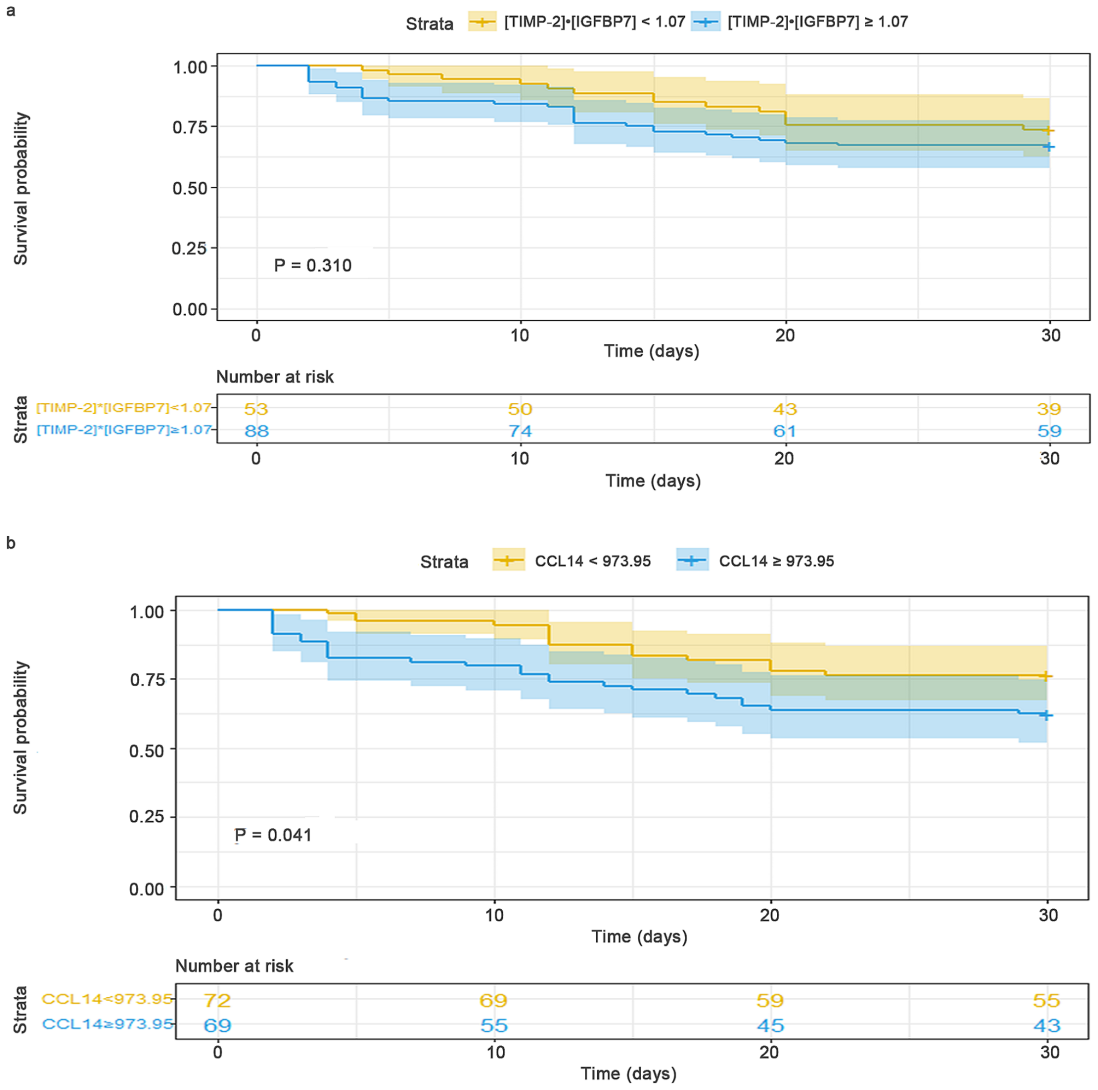
Association of [TIMP-2]•[IGFBP7] and CCL14 with 30-day mortality after SA-AKI. **a**: The mortality rates were not significantly different when the patients were grouped according to urinary [TIMP-2]•[IGFBP7] level that were higher or less than 1.07 (ng/mL)^2^/1000. **b**: The mortality rates were significantly different when the patients were grouped according to urinary CCL14 level that were higher or less than 973.95 pg/mL. *Abbreviations*: TIMP-2, Tissue inhibitor of metalloproteinase-2; IGFBP7, Insulin-like growth factor-binding protein 7; CCL14, C-C motif chemokine ligand 14

The present study examined the potential association between [TIMP-2]•[IGFBP7] and CCL14 levels with 30-day mortality in patients with SA-AKI. Among the SA-AKI cohort of 141 patients, a total of 36 individuals (25.5%) experienced mortality within 30 days following AKI. ROC curves were utilized to compare the predictive value of [TIMP-2]•[IGFBP7] and CCL14 for 30-day mortality following AKI, as presented in Table 6; Fig. 5. The AUC values indicated that the urinary levels of CCL14 and [TIMP-2]•[IGFBP7] exhibited poor predictive power for 30-day mortality after SA-AKI, with AUC values of 0.623 (95% CI, 0.538–0.704) and 0.593 (95% CI, 0.507–0.675), respectively. Based on the cut-off values of [TIMP-2]•[IGFBP7] and CCL14 for predicting renal non-recovery, the Kaplan Meier curve was used to compare 30-day mortality by Log-rank test; only CCL14 ≥ 973.95 pg/mL was associated with a higher risk of 30-day mortality (Fig. 6).

**Table 6:** The areas under the receiver operating characteristic curves for urinary biomarkers to predict 30-day mortality following SA-AKI

Variables	AUC	95% CI	*P*- value
[TIMP-2]•[IGFBP7] ((ng/mL)^2^/1000)	0.593	0.507–0.675	0.108
CCL14 (pg/mL)	0.623	0.538–0.704	0.024

*Abbreviations* AUC, area under the receiver operating characteristic; CCL14, C-C motif chemokine ligand 14; CI, confidence interval; IGFBP-7, insulin-like growth factor-binding protein 7; TIMP-2, tissue inhibitor of metalloproteinases-2.

## Discussion

TIMP-2 and IGFBP7 are indicators of cellular stress expressed in renal tubule cells [22, 23]. Their concentrations increase when the glomerular filtration rate decreases [22], making them reliable biomarkers for AKI’s prediction, diagnosis, and risk stratification [24]. Our previous research indicated the promising potential of urinary [TIMP-2]•[IGFBP7] in predicting non-recovery in critically ill patients with AKI [25]. The investigation conducted by Xie Y et al. focused on the relationship between [TIMP-2]•[IGFBP7] levels and adverse outcomes among ICU patients diagnosed with AKI. The study encompassed all admitted ICU patients 18 years of age or older, with the exclusion of those on maintenance dialysis or those who were anuric upon ICU admission. Utilizing the established [TIMP-2]•[IGFBP7] cutoff value of 0.3 (ng/mL)^2^/1000, which was previously validated in other cohorts, the researchers distinguished patients as having increased [TIMP-2]•[IGFBP7] (+) or not [TIMP-2]•[IGFBP7] (-). Acute kidney injury was identified according to the KDIGO consensus criteria, resulting in patient categorization into four groups: (1) [TIMP-2]•[IGFBP7] (-)/AKI (-), (2) [TIMP-2]•[IGFBP7] (+)/AKI (-), (3) [TIMP-2]•[IGFBP7] (-)/AKI (+), and (4) [TIMP-2]•[IGFBP7] (+)/AKI (+). The study’s outcomes indicated that [TIMP-2]•[IGFBP7] is a viable biomarker for pinpointing AKI patients who are at a heightened risk for requiring KRT or facing mortality within the ICU [26]. This aligns with the findings from our current research regarding the link between [TIMP-2]•[IGFBP7] and the necessity for KRT in an ICU setting. The study by Koyner JL et al. encompassed a cohort of critically ill patients, explicitly excluding those with moderate or severe AKI (KDIGO stages 2 or 3). Outcomes were assessed at the 9-month mark post-enrollment, with patients being categorized based on the occurrence of either death or dialysis, or the absence of both. Findings from the study suggest that the early measurement of [TIMP-2]•[IGFBP7] levels in critically ill individuals can serve as a potential indicator for elevated risk of mortality or the necessity for KRT within the subsequent 9 months. It is important to note that the research did not separately explore the relationship between [TIMP-2]•[IGFBP7] levels and the individual outcomes of death or dialysis. Instead, it examined their link to a combined endpoint - either death or dialysis [27]. The research conducted by Godi I et al. also delved into the prognostic capabilities of [TIMP-2]•[IGFBP7] in determining the risk of short-term adverse outcomes in critically ill patients. The findings indicated that both [TIMP-2]•[IGFBP7] and procalcitonin could be instrumental in identifying ICU patients with a high probability of developing septic AKI and encountering short-term complications [28]. This study took the form of a retrospective cohort analysis, suggesting that while the results are noteworthy, they would benefit from verification in a more extensive, prospective trial. Notably, the scope of this study did not cover the association of [TIMP-2]•[IGFBP7] with renal recovery within 7 days post-AKI or mortality at 30 days after the onset of AKI.

CCL14 is a member of the small-molecule chemokine family and was initially thought to play a crucial role in leukocyte chemotaxis. It has also been associated with tissue damage and repair mechanisms. Elevated levels of CCL14 can lead to the substantial recruitment of monocytes and T cells, which have the potential to differentiate into T1 and M1 macrophage cells within an inflammatory injury setting. These macrophage cells possess pathogenic properties and can incite and amplify tissue damage [26]. Urinary CCL14 demonstrated the highest predictive value for persistent stage 3 AKI among the biomarkers studied [14]. However, in our previous study about AKI in critically ill patients [15], CCL14 did not show a significant advantage over [TIMP-2]•[IGFBP7] in predicting renal non-recovery within a mixed AKI population. Consequently, we aimed to evaluate and compare the predictive capabilities of urinary biomarkers [TIMP-2]•[IGFBP7] and CCL14 for renal non-recovery, the initiation of KRT in the ICU, and 30-day mortality, focusing specifically on a relatively homogeneous population of patients with SA-AKI. 22 stage 2–3 SA-AKI patients reported in the previous publication [15] and additional 119 stage 2–3 SA-AKI patients were included in current study. It was found in this study that urinary CCL14 exhibited superior predictive ability (AUC = 0.901) in comparison to [TIMP-2]•[IGFBP7] (AUC = 0.730) for renal non-recovery.

AKI is a widely prevalent condition within ICU, with approximately 40% of cases typically attributed to sepsis [29, 30]. Among patients with SA-AKI, 40% exhibit moderate to severe AKI, while 27% experience severe AKI, necessitating continuous KRT [31]. The pathophysiology of AKI in critically ill patients is highly heterogeneous, encompassing a variety of underlying causes such as sepsis, surgical interventions, and trauma. In SA-AKI, the pathogenesis is closely associated with the host’s inflammatory and immune responses elicited by the infectious insult. As a result, biomarkers like [TIMP-2]•[IGFBP7] and CCL14 may demonstrate different predictive capacities in the context of SA-AKI compared to other critical illnesses, due to specific pathophysiological dynamics. By focusing on SA-AKI patients, our research seeks to create a more homogenized cohort, especially regarding the causes of AKI. A uniform study population in terms of AKI etiology could improve the accuracy of biomarker performance assessment. Applying this focused method might result in more precise and trustworthy prognostic insights related to renal recovery, as the biomarkers’ effectiveness could be more directly correlated with the distinctive pathophysiology of this patient subgroup. However, the combination of [TIMP-2]•[IGFBP7] and CCL14 did not enhance the predictive performance beyond that of CCL14 alone.

Our study found that urinary [TIMP-2]•[IGFBP7] or CCL14 exhibited moderate predictive capability in anticipating the need for KRT in the ICU among SA-AKI patients. Furthermore, the predictive model that integrated the concentrations of [TIMP-2]•[IGFBP7] and CCL14 demonstrated a commendable capability, as evidenced by an AUC value exceeding 0.8. Additionally, our study revealed that patients with SA-AKI who exhibited urinary CCL14 levels equal to or exceeding 973.95 pg/mL exhibited a greater likelihood of experiencing 30-day mortality. Unfortunately, elevated levels of [TIMP-2]•[IGFBP7] did not demonstrate a significant correlation with an increased risk of 30-day mortality. KRT is an essential strategy in the treatment of SA-AKI. Nevertheless, there is a lack of universally accepted indicators for KRT in patients with renal non-recovery. The optimal timing for initiating KRT is presently a subject of debate, and there is no definitive tool available to determine its use, resulting in significant variability as the decision ultimately lies with the treating physicians. Our current study suggests that incorporating urinary [TIMP-2]•[IGFBP7] and CCL14 may help clinicians in making informed decisions about initiating KRT, which could potentially reduce renal damage and improve survival rates.

The utilization of specific biomarkers provides clinicians with a powerful tool to classify patients based on their risk levels. This advance in early detection empowers healthcare providers to engage in a more rigorous monitoring and to undertake timely interventions, which holds the potential to halt the furtherance of kidney injury. Enhanced monitoring protocols entail more frequent assessment of renal function and meticulous oversight of fluid and electrolyte equilibrium. The timely interventions could encompass the optimization of hemodynamic status, avoidance of nephrotoxic agents, and the possible use of therapeutic interventions that have shown promise in protecting kidney function, such as hydration protocols, antioxidants, or drugs that target specific pathways involved in AKI [32–34]. Furthermore, it grants enhanced clarity in making pivotal decisions regarding the necessity and the optimal moment to commence KRT. In general, the predictive value of these biomarkers for renal non-recovery offers a powerful tool in the management of SA-AKI patients. By enabling early identification, risk stratification, and the initiation of targeted treatments, these biomarkers hold the potential to significantly improve patient outcomes by preventing or delaying the progression of kidney injury.

### Strengths and limitations

This study included stage 2–3 SA-AKI patients with relatively high population homogeneity. However, The study possesses certain limitations. First, further evaluation and validation of urinary [TIMP-2]•[IGFBP7] and CCL14 for renal non-recovery is necessary through extensive sample size studies, despite the inclusion of SA-AKI patients from two ICUs. Second, the lack of continuous monitoring when measuring urinary [TIMP-2]•[IGFBP7] and CCL14 levels solely at the initiation of AKI makes it impossible to determine the changes in [TIMP-2]•[IGFBP7] and CCL14 in SA-AKI patients with renal recovery or non-recovery.

## Conclusion

In the prediction of renal non-recovery in cases of stage 2–3 SA-AKI, urinary CCL14 demonstrated superior predictive capacity compared to [TIMP-2]•[IGFBP7] with an AUC value exceeding 0.9. It’s worth noting that incorporating [TIMP-2]•[IGFBP7] alongside CCL14 did not markedly improve the predictive accuracy over using CCL14 on its own. When it came to predicting the need for KRT in the ICU, both urinary [TIMP-2]•[IGFBP7] and CCL14 showcased moderate prognostic performance. However, our refined predictive model that combined [TIMP-2]•[IGFBP7] with CCL14 displayed a good predictive power for KRT necessity in the ICU, achieving an AUC greater than 0.8.

### Electronic supplementary material

Below is the link to the electronic supplementary material.

Supplementary Material 1

## Data Availability

The datasets analyzed during the current study are available from the corresponding author upon reasonable request.

## Abbreviations

AKI: Acute kidney injury
CCL14: C-C motif chemokine ligand
SA-AKI: Sepsis-associated acute kidney injury
KDIGO: Kidney Disease: Improving Global Outcomes
ICU: Intensive Care Unit
TIMP-2: Tissue inhibitor of metalloproteinase-2
IGFBP7: Insulin-like growth factor-binding protein 7
KRT: Kidney replacement therapy
SOFA: Sequential organ failure assessment
APACHE II: Acute physiology and chronic health evaluation II
SCr: Serum creatinine
BMI: Body mass index
COPD: Chronic obstructive pulmonary disease
SD: Standard deviation
AUC: The area under receiver operating characteristics curve
IQR: Interquartile range
ROC: Receiver operating characteristic
AUROC: Area under the receiver operating characteristic
OR: Odds ratio
CI: Confidence interval
