# Supplementary material for: Predictive performance of two types of urinary biomarkers for renal non-recovery in sepsis-associated acute kidney injury: a prospective observational study
Source: BMC Nephrol. 2024 May 3;25:153. doi: 10.1186/s12882-024-03589-9 (PMC11067266; doi:10.1186/s12882-024-03589-9)
Supplement: Supplementary file 1 — Supplementary Material 1 [file 12882_2024_3589_MOESM1_ESM.doc]

**Supplemental Table S1**

**Delong analysis for the contributions of two kinds of urinary biomarkers for renal non-recovery**

| **Variables** | **Z value** | ***p-*value** |
| --- | --- | --- |
| CCL14 vs [TIMP-2]•[IGFBP7] | 3.637 | 0.001 |
| CCL14 vs [TIMP-2]•[IGFBP7] - CCL14 | 0.467 | 0.641 |
| [TIMP-2]•[IGFBP7] vs [TIMP-2]•[IGFBP7] - CCL14 | 4.574 | <0.001 |

Abbreviations: TIMP-2, tissue inhibitor of metalloproteinases-2; IGFBP7, insulin-like growth factor-binding protein 7; CCL14, C-C motif chemokine ligand 14

**Supplemental Table S2**

**Delong analysis for the contributions of two kinds of urinary biomarkers for prediction of kidney replacement therapy use in ICU**

| **Variables** | ***Z* value** | ***p-*value** |
| --- | --- | --- |
| CCL14 vs [TIMP-2]•[IGFBP7] | 1.202 | 0.230 |
| CCL14 vs [TIMP-2]•[IGFBP7] - CCL14 | 1.009 | 0.313 |
| [TIMP-2]•[IGFBP7] vs [TIMP-2]•[IGFBP7] - CCL14 | 2.123 | 0.034 |

Abbreviations: TIMP-2, tissue inhibitor of metalloproteinases-2; IGFBP7, insulin-like growth factor-binding protein 7; CCL14, C-C motif chemokine ligand 14; ICU, Intensive Care Unit
